# Pathological Condition of the Dental Pulp

**Published:** 1871-12

**Authors:** S. P. Cutler


AMERICAN JOURNAL

OF

DENTAL SCIENCE.

Vol. V. THIRD SERIES--DECEMBER, 1871. No. 8.

### ARTICLE I.

### *Pathological Condition of the Dental Pulp.*

BY S. P. CUTLER, M. D., D. D. S.

What are the probabilities of saving a portion of tooth pulp in a healthy condition, after a portion had sloughed from exposure by decay, and at what point in the pulp chamber would be the most likely to be successful?

I have often witnessed in cases of exposure and partial sloughing after extracting the tooth, that a portion of the pulp included in the fang opens up a certain distance, sometimes nearly the entire length of the fang, that the pulp presented a normal appearance, only slight fullness in the vessels, the sloughing portion being somewhat decomposed with the usual smell; on splitting open the tooth sometimes pus is present.

In many cases of this kind there is scarcely any pain or soreness, the alveolus and membrane normal, more especially in single fang teeth. In such cases, is there not an indication that nature is making a saving or conservative effort to retain a portion of such pulp from total annihilation? If so, is not an attempt on the part of the dentist, to save not only the tooth but a portion of the nerve also, in a healthy condition, proper?

We often find great tenacity to life in this remaining portion of nerve, even after arsenic has been used with partial success, so much so that no subsequent application succeeds in total destruction of the remaining portion. I have often noticed that in such cases, also in spontaneous partial sloughing, that the point diseased was just about the point where the tubuli leave the pulp canal, and terminate at the margin of the enamel, at the junction of cement or about on a line with the alveolus or a little lower.

This fact in itself indicates clearly to my mind the point where we might attempt to save the remaining portion of pulp, owing to the fact that all tubular nerve fibrils below that point ramify through the cement to the periosteum, thereby giving them greater vitality to life than those terminating in the crown, at the more mineral or less vital tissue, the enamel.

There are many cases of decay where exposure of pulp from any cause, produces no pain or uneasiness whatever even where a larger number of pulps are exposed at the same time, and undergo slow and gradual devitalization without any actual sloughing, instead of a drying up and gradual loss of sensibility. In such cases I have noticed a normal condition of the fang portion of remaining pulp, no signs of inflammation or sloughing. Again, on the other hand, and that in a majority of cases, we find inflammation accompanied with pain, followed by sloughing, frequently baffling all attempts to relieve, necessitating extraction sooner or later, or killing of pulp outright, with small assurance of permanent success. In cases of the former variety, we are fully justified in attempting to save such exposed pulps, or even where there is a portion of pulp gone by slow decay, or as Liebig might call it *eremacosis*. I but recently filled such teeth with os-artificiel with promise of success. Carbolic acid, styptic colloid, tannin, and creosote and many other medicaments may be of service before filling. How long such pulps may live or what per cent. of them ossify, we have no definite data to decide the point. Sometimes the bulbous portions of the pulp may be sloughed away and down the fang to midway, and there making a stand as it were, in the "last ditch" for dear life for an indefinite time, sustained and backed by other agencies, such as the hypertrophied or thickened cement up to that point, and then stopping abruptly, and continues to grow or thicken so long as the nerve continues to live, at least so long as there is vitality remaining in pulp within the dentine. At the same time the dentine is undergoing changes, the canal narrowing, the tubuli also narrowing, which in effect narrows down vitality to a certain extent, as there is less need for the usual amount of vitality, the area of nervo-hemal is diminished, hence, nature here proportions her necessities to the new condition of things, never being prodigal with means or ends.

When a tooth as above described remains open and exposed to the irritating influences of oval secretions, the cemental and dental changes must necessarily be aggravated and carried too far or to a point beyond conservative limits and total loss demanded, but where the last stand is made and timely aid arrives the tooth may be saved for an indefinite time, and made serviceable and companionable to the possessor. There is established a dead line in the dentine at the point spoken of, not absolutely so in the cement above this line, as there must be some slight amount of vitality remaining, the dead line being confined to the dentine at the union of cement or thereabouts. One feature I will notice in the process under discussion is this: that when a nerve in a tooth is destroyed wholly or in part, that afterwards there is no growth of cement at all above the point where vitality in the pulp has been destroyed, but may be the case up to the point of neural vitality in the fang, this growth being the effect of irritation in the alveolus and fang, weakening vital resistance to lime encroachments surrounding the fang.

In this process, new improvised germinal matter, as Mr. Beale calls it, is improvised in the periosteum, and then subsequent ossification of these cells of germinal matter or colloid matrix takes place. Now I am only theorizing from want of positive knowledge, and instead of any new cells improvised which are to undergo subsequent ossification, the periosteal cells may take an ossification *per se*, and in consequence of weakened vitality, thereby yielding to ossific degeneration which is a downward process towards the mineral world, from whence all organisms originate. In the first place a thickening of the inter-alveolar membrane takes place and more or less loosening of the tooth takes place in many cases.

Now as the cement ossifies, the inner walls of the alveolus must be carried away to make room for the encroaching growth of cement. This is what is usually called absorption of the bone, which is actually the case, but the question is, how does this absorption take place? as the agencies that break up bone and cause bone absorption, would theoretically prevent the growth of cement, still facts are facts and cannot be ignored. In the above process one side of the membrane forms semi-bone, and the other surface of this membrane must generate an acid of sufficient energy to decompose at least one equivalent of the lime of the tubasic phosphate of lime, leaving only a bi-phosphate which is an acid or superphosphate which is a soluble salt of lime, and is now wholly disorganized and easily removed. The carbonate also being amenable to this acid action which readily disposes of the earthy matter, and the pressure of the encroaching cement causes absorption of the ostiene or cartilage, by the usual process of oxidation. Oxidation is incompetent to remove the earthy elements of bone, as that is already oxidized before it becomes a phosphate and carbonate, and cannot take up or hold any more oxygen, the calcium being completely saturated with it. After the entire nerve is dead and gone from the entire pulp canal, on cutting out the canal with an excavator some slight sensibility, as a general thing is felt at a certain point only, especially where the dentine is exceedingly thin. I operated but the other day on just such a case where spontaneous death of the entire pulp had taken place some time previous. We often, in drilling pivot holes in fangs for pivot teeth, find some sensibility when one drils so as to closely encroach upon the cement. I am more convinced of these facts now than formerly.

Now the tide in the affairs of teeth with only a fang portion of pulp left alive turns, in my cordial opinion, upon the fact whether or not this remaining portion takes on secondary ossification? If so all may go well if not too much hurried. When this process fails, the probability is that in most cases death ensues no matter what method is pursued. We have not facts enough before us to predicate a percentum, but I here beg leave again to ask, are not the facts already before us sufficient to justify us under favorable circumstances to attempt to save such pulp alive in preference to any other course? I certainly hear an echo saying yes.

I will not pretend to advise any particular method how to treat or fill even in such cases, only give my favorite method: I prefer as an immediate covering over the pulp after proper treatment, gutta-percha dissolved in chloroform, as it readily settles down over the pulp without any pressure or imitation and readily evaporates, leaving a layer of any desirable thickness over the tissue. I have sometimes used os-artificiel, but this is more of a chemical agent than I like just here, and may be added after the gutta-percha, and then anything else the operator may prefer. I have had some experience in this direction and some success, though not extensive. We need more post mortem dental data on the subject.

